# Gas6 Induces Myelination through Anti-Inflammatory IL-10 and TGF-β Upregulation in White Matter and Glia

**DOI:** 10.3390/cells9081779

**Published:** 2020-07-26

**Authors:** Salman Goudarzi, Shannon E. Gilchrist, Sassan Hafizi

**Affiliations:** School of Pharmacy and Biomedical Sciences, University of Portsmouth, Portsmouth PO1 2DT, UK; salman.goudarzi@port.ac.uk (S.G.); shannon.gilchrist@port.ac.uk (S.E.G.)

**Keywords:** IL-10, TGF-β, Gas6, TAM receptor, optic nerve, vitamin K, microglia, astrocytes, myelination, white matter

## Abstract

The Gas6–TAM (Tyro3, Axl, Mer) ligand–receptor system is believed to promote central nervous system (CNS) (re)myelination and glial cell development. An additional important function of Gas6–TAM signalling appears to be the regulation of immunity and inflammation, which remains to be fully elucidated in the CNS. Here, we characterised the expression of TAM receptors and ligands in individual CNS glial cell types, observing high expression of Gas6 and the TAM receptors, Mer and Axl, in microglia, and high expression of Tyro3 in astrocytes. We also investigated the effect of Gas6 on the inflammatory cytokine response in the optic nerve and in mixed glial cell cultures from wildtype and single TAM receptor knockout mice. In wildtype and Mer-deficient cultures, Gas6 significantly stimulated the expression of the anti-inflammatory/pro-repair cytokines interleukin 10 (IL-10) and transforming growth factor β (TGF-β), whereas this effect was absent in either Tyro3 or Axl knockout cultures. Furthermore, Gas6 caused upregulation of myelin basic protein (MBP) expression in optic nerves, which was blocked by a neutralising antibody against IL-10. In conclusion, our data show that microglia are both a major source of Gas6 as well as an effector of Gas6 action in the CNS through the upregulation of anti-inflammatory and pro-repair mediators. Furthermore, the presence of both Axl and Tyro3 receptors appears to be necessary for these effects of Gas6. In addition, IL-10, alongside suppressing inflammation and immunity, mediates the pro-myelinating mechanism of Gas6 action in the optic nerve. Therefore, Gas6 may present an attractive target for novel therapeutic interventions for demyelinating as well as neuroinflammatory disorders of the CNS.

## 1. Introduction

The rapid recognition of living and dead cells in order to clear cell debris is important for the maintenance of the central nervous system (CNS) microenvironment and for immunological tolerance and resolution of inflammation. Microglia, the resident immune cells of the CNS, contribute in a major way to the regulation of inflammation [1]. They function as a crucial defence against various types of insult to tissues and cells within the brain [2,3], with the ability to exist in either a resting state (ramified) or an active state (amoeboid) [4]. In the case of inflammation, infection or trauma, microglia rapidly convert into an activated state and become phagocytic [3]. Also, whilst in an active state, microglia can secrete both pro- and anti-inflammatory cytokines, the latter including interleukin 10 (IL-10) and transforming growth factor β (TGF-β) [4,5]. The anti-inflammatory cytokines are produced in order to antagonise the pro-inflammatory and immune responses in the CNS [5,6,7]. The anti-inflammatory functions of IL-10 include blocking the activity of Th17 cells and inhibiting the production of pro-inflammatory cytokines such as TNF-α, IL-1 and IFN-γ [8]. During the resolution phase of inflammation, TGF-β is upregulated, which enables the draining of leukocytes, tissue repair [9,10,11], as well as myelination [12]. Upon upregulation, through binding to TGF-β receptors and subsequent phosphorylation of Smad receptors, TGF-β eventually contributes to the suppression of inflammation [9,13,14].

We and others have previously shown that the vitamin K-dependent protein Gas6 is an important modulator of cellular processes also in the CNS, such as cell survival, inflammation, proliferation, migration and removal of apoptotic cells and debris [15,16,17]. Gas6 is a ligand for the TAM (Tyro3, Axl, Mer) receptor tyrosine kinases (RTKs) [15,16,17]. Of major significance is also that Gas6–TAM signalling appears capable of regulating the immune system, including the activity of phagocytes such as macrophages and microglia. TAM triple-receptor-deficient mice suffer from a lymphoproliferative disorder that is accompanied by broad-spectrum autoimmunity [17,18]. TAM signalling has also been shown to play a role in oligodendrocyte survival [19] as well as to enhance myelination in a cuprizone model of demyelination [20]. Direct administration of Gas6 into the CNS was protective in experimental autoimmune encephalomyelitis, whilst deletion of Gas6 enhanced inflammation and delayed recovery [21]. In addition, we have reported that Gas6–TAM signalling can protect from demyelination in an ex vivo demyelination model [15]. These findings indicate Gas6 has the ability to negatively regulate neuroinflammation and the immune response as well as induce brain regeneration, all of which make it a potential therapeutic target for relevant disorders such as multiple sclerosis (MS). In the present study, we show that Gas6 exerts anti-inflammatory and pro-repair roles through upregulation, respectively, of IL-10 and TGF-β in the mouse optic nerve and in mixed glial cell cultures.

## 2. Materials and Methods

### 2.1. Animals

Wildtype and Tyro3^−/−^, Axl^−/−^ and Mer^−/−^ C57/BL6 mice, aged postnatal day 0 to 2 (P0–2), were used for mixed and primary glial cultures, whereas adult, 2-month old, mice were used for ex vivo experiments with optic nerve cultures. The three TAM knockout strains were obtained from The Jackson Laboratories, Bar Harbor, ME, USA. All experiments involving animals were performed in accordance with the Home Office Animals (Scientific Procedures) Act 1986 under a UK Home Office project licence (licence number PC2238199; 22/8/2017), as well as following approval from the University of Portsmouth ethics review committee (AWERB). Animals were killed humanely by cervical dislocation, and tissues were removed rapidly and placed in ice-cold saline prior to experiments.

### 2.2. Optic Nerve Culture

Mouse optic nerve cultures were set up for incubation over several days as previously described [22]. Briefly, optic nerves with eyeball attached were removed from 2-month-old mice and placed in ice-cold artificial cerebrospinal fluid containing 124 mM NaCl, 2.5 mM KCl, 26 mM NaHCO_3_, 2 mM MgSO_4_, 1.25 mM KH_2_PO_4_, 10 mM glucose, 4 mM sucrose, 2.5 mM CaCl_2_, pH 7.4. The tissue was placed onto semi-porous membrane inserts (0.4 mm; Millipore, Watford, UK) in a six-well culture plate and covered with 2 mL culture medium consisting of 50% Opti-MEM, 25% Hanks Balanced Salt Solution, 25% horse serum (Gibco Invitrogen, Paisley, UK), supplemented with 25 mM D-glucose (Sigma, Gillingham, UK) and antibiotics (penicillin G sodium 10,000 U/mL, streptomycin sulphate 1000 mg/mL; Gibco Invitrogen) at a 1:500 dilution. For experiments, recombinant Gas6 protein [15] was added directly to the culture medium to a final concentration of 400 ng/mL; culture medium alone was used as control treatment. Treatments were replenished on the second day of incubation. After 3 days, optic nerves were detached from the eye, and the tissue was processed for subsequent applications. All experiments were repeated using cultures prepared from different animals at different times.

### 2.3. Cell Culture

#### 2.3.1. Primary Mixed Glial Cell Culture

Mixed glial cell cultures were established from mice at postnatal ages P0–2 according to the method described by Mecha et al. [23]. Briefly, after dissection of the brain and removal of the brain stem and cerebellum, the forebrain was placed into cold Dulbecco’s modified eagle medium (DMEM), and the meninges were removed under a dissecting microscope (VWR, Lutterworth, UK). The forebrain was transferred into a 50 mL falcon tube containing 1 mL cold DMEM, where it was triturated and dissociated using a serum-coated glass Pasteur pipette. The tube was centrifuged at 168× *g* for 10 min. The pellet was re-suspended in warm DMEM 10:10:1 containing 10% foetal calf serum (FCS), 10% horse serum and 1% penicillin/streptomycin. Re-suspended cell pellets were added to tissue culture flasks or 24-well plates pre-coated with poly-D-lysine (Sigma). Cells were incubated at 37 °C in a humidified atmosphere of 5% CO_2_ in air for minimum 10 days. Microscopic analysis of the cultures verified a consistent proportion of astrocytes/microglia across the various cultures. Following the incubation setup period, the cells were processed either for setting up pure glial cell cultures or for direct use in experiments, treating with recombinant Gas6, lipopolysaccharide (LPS, 10 ng/mL; Sigma) or medium only. LPS is a Gram-negative bacterial cell wall component that can stimulate the production of a number of inflammatory cytokines in various cells. Following 24 h of incubation, the cells were processed for subsequent applications.

#### 2.3.2. Primary Pure Glial Cell Culture

##### Microglia

Following isolation and culture of mixed glial populations over 10 days as described above, separation of distinct glial cell types was performed as described by Mecha et al. [23]. Briefly, flasks containing settled mixed glial cultures were shaken orbitally at 230 rpm for 3 h at 37 °C. The suspension of cells following shaking, containing detached microglia, was collected and immediately added to warm DMEM 10:10:1 in tissue culture flasks. The cell suspension was centrifuged for 10 min at 168× *g*, the cell pellet was resuspended in warm DMEM 10:10:1, and cells were added to a 24-well plate and incubated at 37 °C in a humidified atmosphere of 5% CO_2_ in air. Immunofluorescence analysis of cultures for distinct cell type markers, Iba1 (microglia), GFAP (astrocytes), Sox10 (oligodendrocyte precursor cells, OPCs), verified their identity and purity at ≥95%. The cells then underwent various treatments for experiments, as described in the Section Results.

##### Astrocytes

According to the method described by Mecha et al. [23], following the initial detachment of microglia from the mixed glial cultures, the remaining cells were placed on an incubating orbital shaker and shaken at 260 rpm overnight at 37 °C. The following day, the cell suspension, mostly containing oligodendrocyte-lineage cells, was removed, and the remaining attached astrocytes were detached from the culture plastic surface by tryptic digestion (TrypLE^™^ Express; Gibco Invitrogen). The cell pellet following centrifugation was resuspended in warm DMEM 5:5:1 containing 5% FCS, 5% horse serum and 1% penicillin/streptomycin. Cells were added to a 24-well plate and incubated at 37 °C in a humidified atmosphere of 5% CO_2_ in air. As stated above, immunostaining analysis verified the cultures’ identity and purity at ≥95%. The cells then underwent various treatments for experiments, as described in the Section Results.

### 2.4. Reverse-Transcription Quantitative PCR

Total RNA was isolated from cultured optic nerves as well as cultured mixed glial cells using a total RNA extraction kit (RNeasy Mini Kit; Qiagen, Manchester, UK). RNA was reverse-transcribed (Applied BioSystems, Loughborough, UK) to produce cDNA for subsequent real-time quantitative polymerase chain reaction (qPCR) analysis. qPCR analysis was performed using specific primers and fluorescent hydrolysis probes for each gene (Integrated DNA Technologies; Leuven, Belgium) within a reaction master mix (FastStart Essential DNA Probes Master; Roche, Burgess Hill, UK). The expression levels of all genes in the samples were normalised against the expression of the mouse *Gapdh* gene as reference gene. All samples were analysed based on the ΔC_T_ and ΔΔC_T_ method, where ΔC_T_ is C_T_[Target gene]-C_T_[Housekeeping gene], and 2^−ΔCT^ represents the relative gene expression. ΔΔC_T_ is ΔC_T_[Gas6]-ΔC_T_[Mock], and 2^−ΔΔCT^ shows fold up- or downregulation of the gene of interest, whereby values >1 and <1 are deemed as corresponding to up- and downregulation, respectively [24].

### 2.5. SDS-PAGE and Western Blot

Total protein was extracted from cultured optic nerves for SDS-PAGE analysis of proteins. Tissues were homogenised in lysis buffer composed of 50 mM Tris-HCl, 150 mM NaCl, 1% Triton X-100, 0.5% NP-40, 1 mM EDTA, 10 mM Na_4_P_2_O_7_, pH 8.0. Equal amounts of total protein were loaded onto a 10% polyacrylamide gel, and proteins were separated by SDS-PAGE as previously described [25]. The separated proteins in the gel were transferred to a polyvinylidene fluoride membrane (Immobilon-P; Millipore, Watford, UK). Membranes were first blocked in 3% non-fat dry milk in 25 mM Tris, 150 mM NaCl, 0.05% Tween-20, pH 8.0, for 1 h at room temperature (RT), after which they were incubated with primary antibodies at 4 °C overnight. Primary antibodies were against β-actin (dilution, 1:10000, A2066; Sigma) and myelin basic protein (MBP, dilution, 1:500, MAB386; Millipore, Darmstadt, Germany). Washing of the membrane (25 mM Tris-HCl, 150 mM NaCl, 0.05% Tween-20, pH 8.0) was followed by 1 h incubation with a horseradish peroxidase-conjugated secondary antibody recognising the appropriate primary antibody (1:5000; Promega, Southampton, UK; Dako, Glostrup, Denmark) at RT. A chemiluminescence detection reagent (Luminata Forte Western HRP Substrate; Millipore) was used to develop the signal, and bands were visualized by a CCD-based gel imager (Bio-Rad ChemiDoc^™^ MP, Hemel Hempstead, UK). Band intensities were quantified by densitometry using ImageJ software, with band intensities of the proteins of interest being normalised against those of actin protein bands in every sample lane.

### 2.6. IL-10 ELISA

The cell culture supernatant was collected from mixed glial cultures following experimental treatments as described in the Section Results, and samples were stored at −80 °C. ELISA for mouse IL-10 was performed in duplicate wells per each sample, following the manufacturer’s instructions (mouse IL-10 ELISA kit, Bio-Techne, Abingdon, UK).

### 2.7. Statistical Analysis

All statistical analyses were performed using the software Prism 6 (GraphPad Inc, La Jolla, CA, USA). Animal tissue cultures were randomly assigned to the treatment groups. All results are expressed as mean ± SEM, with each experiment performed a minimum 3 times, as specified in the figure legend, using multiple replicates per treatment. Differences between multiple treatments in mixed glial cultures were compared using paired parametric student *t* test and, in optic nerve cultures, were compared using unpaired parametric *t* test. A *p* value of less than 0.05 was considered statistically significant.

## 3. Results

### 3.1. The Expression of TAM Receptors and Gas6 in Different Glial Cell Types

**Figure 1:**
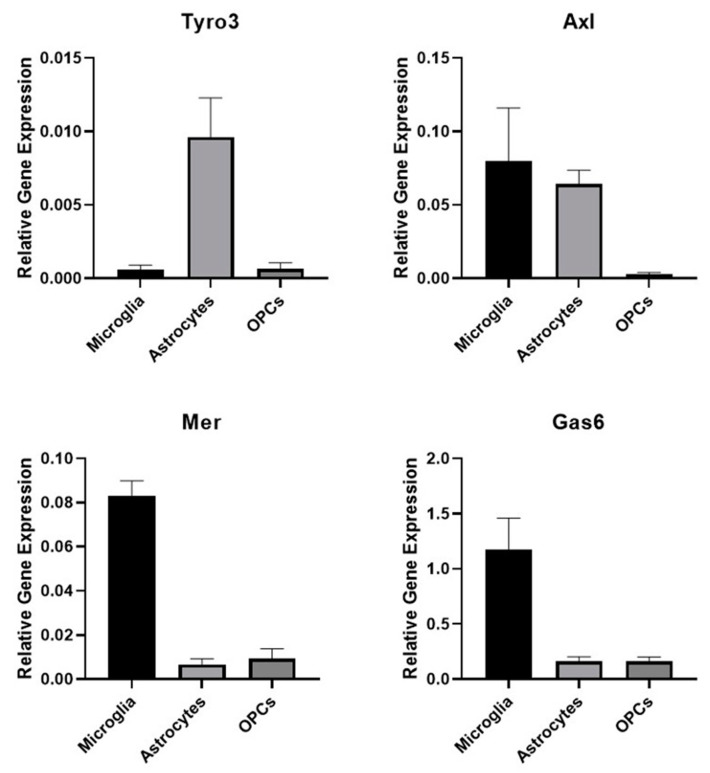
Expression of TAM (Tyro3, Axl, Mer) receptors and Gas6 in pure primary cultures of microglia, astrocytes and oligodendrocyte precursor cells (OPCs). Quantitative RT-PCR analysis of mRNA expression of the genes for Tyro3, Axl, Mer and the ligand Gas6 in extracts from mouse primary glial cell cultures. Values represent mean ± SEM (*n* = 3 experiments from different mice for all samples).

We performed qRT-PCR analysis to first establish the TAM expression profiles in the cultures of pure mouse primary glial cell types. Amongst the cultures, only astrocytes showed detectable expression of Tyro3 and of Axl (Figure 1). Microglia showed strong expression of Mer and Axl receptors as well as of the TAM ligand Gas6. OPCs showed negligible expression of all genes analysed.

### 3.2. Gas6 Increases IL-10 Expression in Optic Nerve Via Axl and Tyro3 Receptors

**Figure 2:**
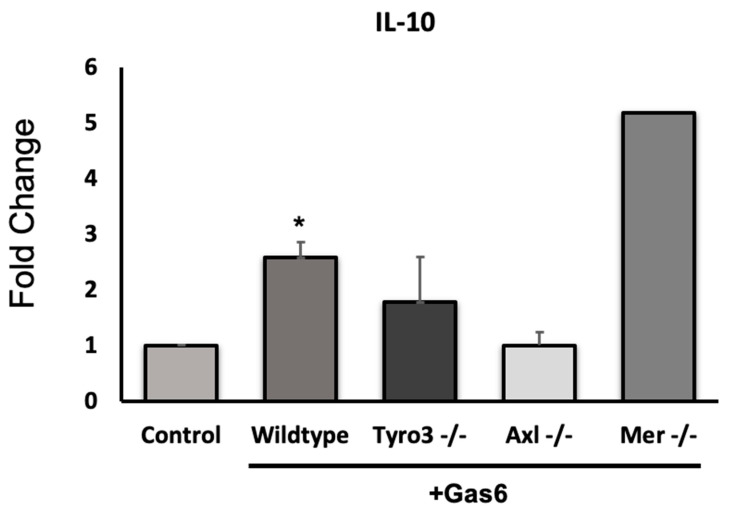
Effect of Gas6 on interleukin 10 (IL-10) gene expression in cultured mouse optic nerve. qRT-PCR was performed on extracts from optic nerves using specific *il10* primers/probe, using *Gapdh* as reference gene. Gas6 caused the upregulation of the *il10* gene only in wildtype and Mer knockout optic nerves. Values represent mean ± SEM (*n* = 4 experiments for wildtype (* *p* < 0.05 vs. control); *n* = 3 for Tyro3^−/−^ and Axl^−/−^; *n* = 2 for Mer^−/−^). The ‘Control’ bar represents a normalised value based on every experiment for each mouse tissue experiment having its own untreated sample.

The effect of Gas6 on the expression of 84 different MS-related genes was analysed in the adult optic nerve using a PCR array (Qiagen). As we reported previously, a set of genes were identified as being significantly altered by Gas6 treatment [15]. These included the genes for matrix metalloproteinase-9 (MMP9), the astrocyte marker GFAP and the RTK Epha1, all of which were downregulated. The gene for the anti-inflammatory cytokine IL-10 was amongst those that were upregulated by Gas6. Therefore, further qRT-PCR analysis of individual genes was performed to confirm the array data. As expected, IL-10 was significantly upregulated in the adult optic nerve in response to Gas6 treatment (Figure 2), as were, for validation by comparison, a set of other genes that we had also previously shown to be downregulated by PCR array. In order to identify the TAM receptor that mediated the effect of Gas6 on IL-10, we conducted qPCR analysis on Gas6-treated optic nerve cultures from Tyro3^−/−^, Axl^−/−^ and Mer^−/−^ mice. We observed that the Gas6-induced increase in IL-10 gene expression was absent in Tyro3- and Axl-deficient optic nerves, whilst it still occurred in Mer-deficient optic nerves (Figure 2; Mer knockout data (fold increase by Gas6): expt (1): 1.315; expt (2): 9.03). This suggests that the presence of both Tyro3 and Axl receptors is necessary for the IL-10-inducing effect of Gas6, whereas the presence of only one of them is not sufficient.

### 3.3. Gas6 Upregulates IL-10 and IL-10R Expression in Mixed Glial Cell Cultures

**Figure 3:**
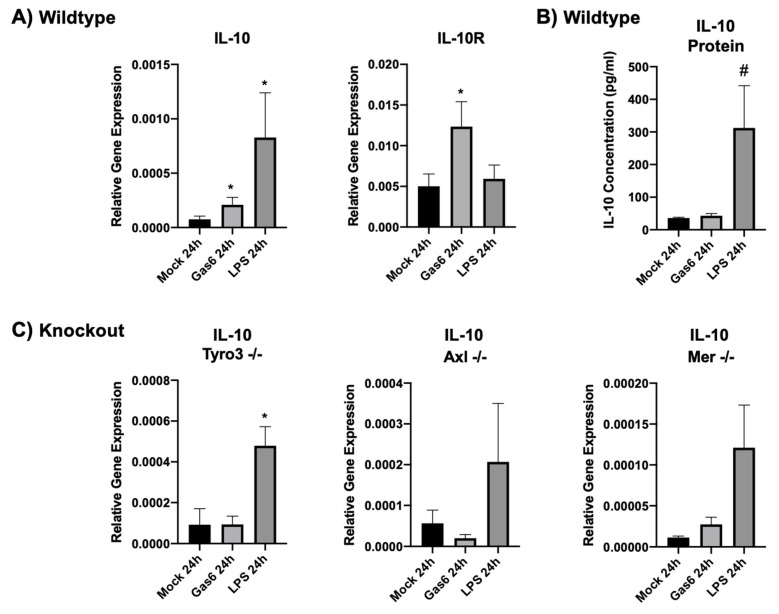
Effect of Gas6 on IL-10 and IL-10 receptor (IL-10R) expression in primary mixed glial cell cultures at gene and protein levels. (**A**) qRT-PCR analysis was performed on extracts from wildtype cultures treated with control, Gas6 and lipopolysaccharide (LPS). Values represent mean ± SEM (*n* = 7 for IL-10, *n* = 6 for IL-10R). (**B**) Mouse IL-10 ELISA was performed on the medium of wildtype mixed glial cultures under the same experimental treatments as in (**A**). Values represent mean ± SEM (*n* = 6 experiments); # *p* = 0.08 vs. control. (**C**) qRT-PCR analysis on extracts from mock-, Gas6 and LPS-treated TAM single-receptor knockout mixed glial cell cultures. Values represent mean ± SEM (*n* = 3); * *p* < 0.05 vs. control.

Having observed that Gas6 upregulates IL-10 in intact optic nerves, we next examined the Gas6 effect in primary glial cell cultures. We had initially determined by qRT-PCR that baseline IL-10 expression in wildtype and TAM knockout microglia was equally very low to negligible (Supplementary Figure S1). Mixed glial cultures were exposed to Gas6 as well as to the pro-inflammatory bacterial cell wall component LPS for comparison and incubated for various times ranging from 1 to 72 h. Gene expression of both IL-10 and IL-10 receptor (IL-10R) was measured by qRT-PCR. The optimal time period of gene induction was determined to be 24 h. Both Gas6 and LPS significantly increased IL-10 gene expression in wildtype glial cells after 24 h, whereas only Gas6 significantly upregulated IL-10R (Figure 3A).

In addition, to identify the specific TAM receptor(s) involved in regulating IL-10 expression, mixed glial cell cultures from TAM single-receptor knockout mice were also subjected to Gas6 and LPS treatments for 24 h, followed by qRT-PCR analysis. In both Tyro3- and Axl-knockout mixed glial cells, Gas6 was no longer able to alter IL-10 expression, whereas in Mer-knockout cells, Gas6-induced IL-10 upregulation was observed to be at about the same level as in wildtype cells, close to significance (Figure 3C). Therefore, in mixed glial cell cultures, exactly as observed in optic nerve cultures, the presence of both Tyro3 and Axl receptors appeared to be necessary for the IL-10-inducing effect of Gas6, but not the presence of only one of them. IL-10 ELISA analysis of the mixed glial culture medium showed that LPS stimulation caused an increase in IL-10 protein in the medium of wildtype cells, whereas Gas6 induced no significant change (Figure 3B).

### 3.4. Gas6 Upregulates TGF-β in Mixed Glial Cell Cultures

**Figure 4:**
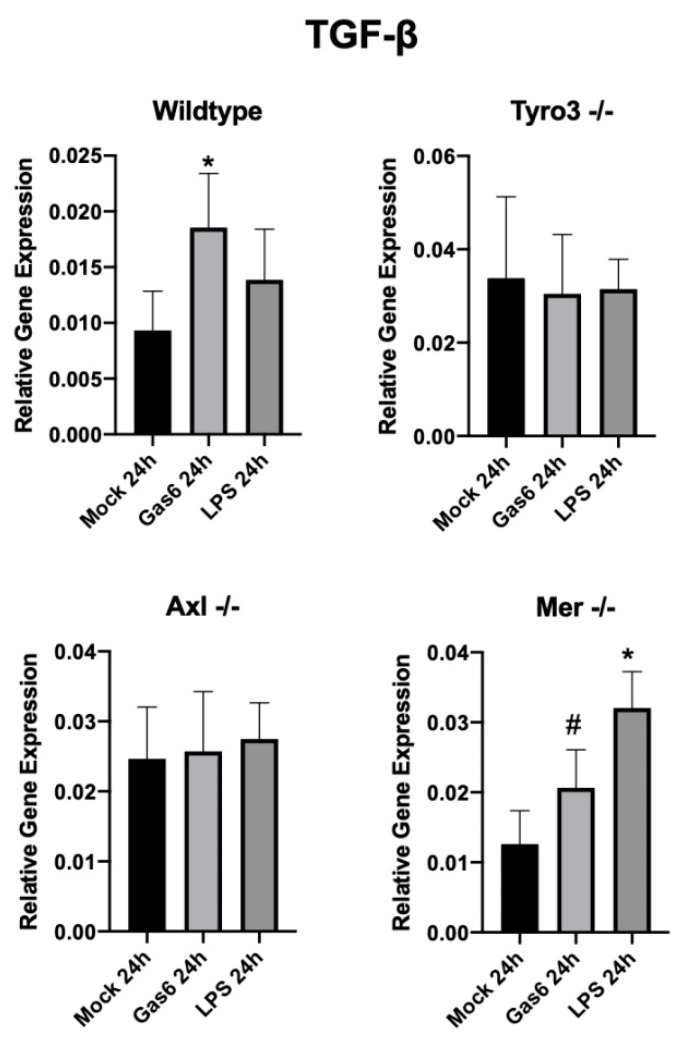
Effect of Gas6 on transforming growth factor β (TGF-β) gene expression in mixed glial cell cultures, measured by qRT-PCR analysis. TGF-β expression in wildtype and single-TAM-knockout primary mixed glial cell cultures, following mock, Gas6 or LPS treatment. Values represent mean ± SEM (*n* = 5 experiments for wildtype, *n* = 3 experiments for TAM knockouts); * *p* < 0.05 and # *p* = 0.08 vs control.

The effect of Gas6 on the expression of the anti-inflammatory and pro-repair cytokine TGF-β was also investigated in mixed glial and optic nerve cultures by qRT-PCR analysis. A TGF-β ELISA was also attempted; however, the levels of protein diluted in the culture medium were too low for reliable measurements (not shown). qRT-PCR analysis showed that Gas6 significantly upregulated TGF-β expression after 24 h in mixed glial cell cultures from wildtype mice (Figure 4). Furthermore, the stimulatory effect of Gas6 was replicated in Mer single-receptor knockout mouse mixed glial cell and optic nerve cultures, but was absent in those from either Tyro3 or Axl single-receptor knockout mice (Figure 4; Supplementary Figure S2). Therefore, as with the regulation of IL-10 expression, the combined presence of both Tyro3 and Axl receptors appeared to be necessary for the regulation of glial TGF-β expression by Gas6.

### 3.5. Gas6 Upregulates MBP in Optic Nerves Via Autocrine IL-10

**Figure 5:**
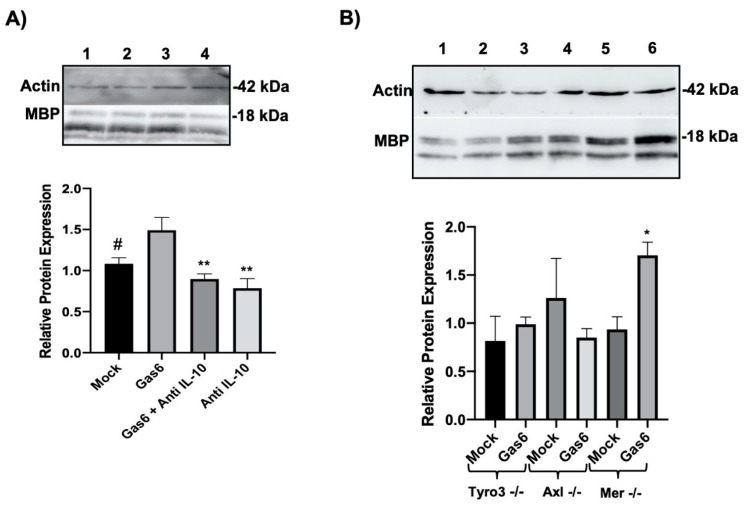
Effect of Gas6 on myelination through myelin basic protein (MBP) expression in cultured optic nerves, analysed by western blotting. Cultured optic nerves were incubated with agents for 3 days. The graphs show the band densitometric quantification of MBP protein levels relative to β-actin protein in each sample. (**A**) Wildtype optic nerves in culture were incubated with Gas6 in the absence or presence of anti-IL-10 antibody. Lanes correspond to the following: (1) Mock, (2) Gas6, (3) Gas6+anti-IL-10, (4) anti-IL-10. Values represent mean ± SEM (*n* = 4 experiments for all the treatments except for the anti-IL-10 group; *n* = 3 experiments); ** *p* < 0.01, # *p* = 0.052 vs. Gas6; no significant difference between Mock and the anti-IL-10 treatments; analysis of variance followed by Dunnet’s multiple comparison. (**B**) Effects of Gas6 on MBP protein expression in TAM single-receptor knockout mouse optic nerve cultures. Lanes correspond to the following: (1) Tyro3^−/−^ mock-treated, (2) Tyro3^−/−^ Gas6-treated, (3) Axl^−/−^ mock-treated, (4) Axl^−/−^ Gas6-treated, (5) Mer^−/−^ mock-treated, (6) Mer^−/−^ Gas6-treated. Values represent mean ± SEM (*n* = 3 experiments); * *p* < 0.05.

We previously reported that Gas6 significantly upregulated MBP expression at gene and protein levels in the optic nerve, concomitant with stimulating STAT3 phosphorylation [15]. Therefore, having observed here that Gas6 also induced IL-10 upregulation in the optic nerve, we investigated whether the two mechanisms are linked and, specifically, whether the Gas6 myelinating effect is mediated through its induction of IL-10. Therefore, we measured MBP at protein level in optic nerves treated with Gas6 in the presence of an anti-IL-10 antibody to potentially neutralise autocrine IL-10. Our western blot data revealed that the upregulation of MBP protein expression by Gas6 was eliminated in the presence of anti-IL-10 neutralising antibodies (Figure 5A). In addition, having observed the role of Tyro3 and Axl in IL-10 upregulation via Gas6, we analysed whether the same signalling mechanism is involved in MBP upregulation in response to Gas6. Therefore, we analysed changes in MBP protein expression in TAM knockout optic nerves treated with Gas6. In the absence of both Tyro3 and Axl, no change in MBP protein level was induced by Gas6, whereas Gas6 was able to upregulate MBP expression in Mer-deficient optic nerves. These data indicate that MBP upregulation is occurring via both Tyro3 and Axl and is subsequent to IL-10 upregulation (Figure 5B).

## 4. Discussion

Previously, we and others have shown that all three TAM receptors are expressed in CNS tissues with distinct expression profiles and that there is a postnatal increase in Tyro3 and Axl expression concomitant with the timeline of myelination [15,26]. However, the involvement of the individual TAM receptors in the biology of specific CNS glial cells remains to be comprehensively characterised. Here, we have determined the expression of Gas6 and the TAM receptors in mouse primary pure glial cell cultures incorporating astrocytes, microglia and OPCs. Also in this study, we have probed further the molecular mechanisms behind the action of Gas6 in the mouse optic nerve, which we previously showed to respond to Gas6 with an increase in OPC numbers, MBP expression and myelination [15]. We observed here that the pro-myelinating effect of Gas6 in the optic nerve occurs through the induction of the anti-inflammatory cytokine IL-10. This effect was reproduced using mixed glial cell cultures, and experiments with TAM single-knockout cells revealed the necessity of both Tyro3 and Axl receptors mediating in concert the Gas6 effect. These results therefore suggest that the recognised role of Gas6–TAM signalling in regulating inflammation is linked to its role in CNS myelination.

The expression of TAM receptors in various regions of the CNS has previously been reported, with Tyro3 being the most prominent TAM overall [26]. Here, we observed that microglia strongly expressed Mer and Axl receptors at mRNA level, whereas astrocytes expressed Axl and appeared to be the only glial cells showing Tyro3 expression. In contrast, OPCs had negligible expression of all these genes. We did also attempt Western blot and flow cytometry as approaches for protein detection in the glial cultures; however, these primary cells were not amenable to efficient protein extraction or did not supply sufficient yields for flow experiments (data not shown). The mRNA expression analysis also revealed microglia to be the main glial cell source of Gas6, showing strong expression. These findings are largely in line with other observations, though with some differences. For example, our observation of Axl expression in microglia contrasts with that of another study which showed Axl expression to be weak [27]. This, however, may be due to differences in the level of expression captured at the time of analysis, tissue source, as well as age. We have shown previously that TAM expression increases during postnatal development through to adulthood [15]. A separate study also showed that Tyro3 expression begins to increase shortly after birth, significantly augmenting until reaching its peak at P24 [26]. Therefore, this can explain the overall negligible expression of all TAMs and Gas6 in our neonatal OPC cultures. Nevertheless, the combined findings of these studies show that TAMs appear to be important in the CNS after birth and, moreover, appear to play distinct roles in different glial cells.

IL-10 is a well-known immunoregulatory cytokine that acts on both haematopoietic and non-haematopoietic cells to control inflammatory responses and immune reactions, also within the brain [28,29]. Amongst its effects is the suppression of secretion of proinflammatory cytokines by monocytes, macrophages and, likely, microglia [28,30,31]. We report here that Gas6 significantly upregulated IL-10 gene expression in cultured optic nerve, which was replicated in cultured mixed glial cells. However, the effect of Gas6 on IL-10 at the protein level as measured by ELISA was not significant; this is most likely due to the low overall protein levels detected in the culture medium and to factors including mRNA translation rate, protein secretion per cell, sufficient cell numbers in the sample, degree of dilution in the medium. Moreover, the intercellular communication model we propose involves IL-10 signalling between closely associating cells in the tissue, and hence, small changes in IL-10 protein concentration in the culture medium may not properly reflect the more impactful in situ role of IL-10. This new finding supports the anti-inflammatory, pro-myelinating and pro-repair role that Gas6–TAM signalling is thought to mediate in the CNS and is in keeping with our previous report of Gas6 upregulation of IL-13 and concomitant downregulation of MMP-9, EphA1 and GFAP genes in the optic nerve [15]. It is also in keeping with the finding that Gas6 increased IL-10 expression in macrophages and was itself stimulated by IL-10 [32].

In addition, we observed that Gas6 stimulated an increase in TGF-β expression in both optic nerve and mixed glial cell cultures. Interestingly, a link between IL-10 and TGF-β signalling in regulating CNS inflammation has been reported, whereby IL-10 released by microglia during inflammation acts on astrocytes to stimulate the latter’s release of TGF-β, which then can act on microglia to suppress inflammation [29]. Furthermore, we used tissue/cells from TAM single-receptor knockout mice in order to identify the specific TAM receptor(s) mediating Gas6 response. Gas6-mediated upregulation of IL-10 and TGF-β expression was abolished in both Axl- and Tyro3-deficient models, whereas it still occurred in the absence of Mer. Therefore, it appears that the joint presence of both Axl and Tyro3 receptors is necessary for Gas6 to be able to exert its anti-inflammatory and pro-repair functions through IL-10 and TGF-β induction. Although the molecular mechanism for this arrangement is not yet known, we speculate that it may involve heterodimerisation amongst these two TAMs and/or a parallel signalling via the separate TAMs that may be most efficacious when combined [33,34,35]. In support of this, in Rat2 cells, Tyro3 and Axl were shown to be closely associated with the ability to cross-phosphorylate and enhance one another’s activation [36].

Considering the fact that both optic nerve and mixed glial culture models contain glial cells, it is most likely that microglia are the main source of IL-10, including in response to Gas6. We also added Gas6 directly to pure microglial cultures; however, no significant increase in IL-10 expression was observed (data not shown), which may suggest an indirect cellular pathway of Gas6 ligand stimulation. In experiments where conditioned medium from astrocytes treated with Gas6 was added to microglia, a slight but insignificant increase in IL-10 expression was observed (data not shown). These supplementary observations therefore indicate that a full effect of Gas6 on IL-10 induction requires a direct physical interaction between microglia and astrocytes, as afforded in the optic nerve and mixed glial cell culture models. Moreover, a crucial role for astrocytes in this mechanism is also indicated by the fact that the presence of Tyro3 is necessary for the Gas6 effect, this TAM receptor being appreciably expressed only in astrocytes.

**Figure 6:**
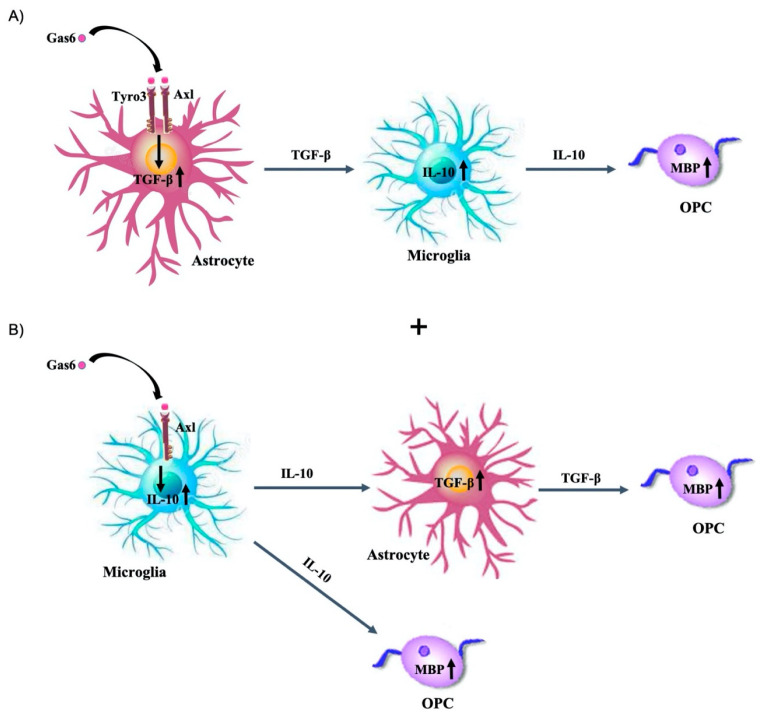
Schematic diagram of two possible mechanisms through which Gas6 exerts its effect on glial cells through IL-10 and TGF-β induction. (**A**) Gas6 indirectly upregulates IL-10 via TGF-β. In this pathway, Gas6 activates astrocytes to release TGF-β which then acts on microglia to stimulate IL-10 production and contributes to the enhancement of myelination. (**B**) In addition, Gas6 could activate IL-10 in microglia via Axl prior to TGF-β upregulation, which then could enhance myelination, as well as stimulate TGF-β in astrocytes. This pathway could occur in the different cell types in parallel; in this case, TGF-β is indirectly activated via IL-10.

We previously reported that Gas6 significantly upregulated MBP expression in the optic nerve and protected against experimental demyelination in brain slice cultures [15]. Moreover, Gas6 has been shown to be capable of boosting remyelination in a cuprizone-induced demyelination model [20]. In addition to its primary role as an anti-inflammatory cytokine, IL-10 may also be involved in CNS regeneration and myelination, as it has been shown to boost myelination and to reduce scar formation in a rodent nerve repair model [37]. Our data indicate an indirect effect of Gas6 on MBP via IL-10 induction, although the intercellular signalling mechanism behind this effect requires full characterisation. One model is that Gas6 stimulates first Tyro3 and Axl receptors on astrocytes, causing the release of TGF-β from these cells, which subsequently acts on microglia to cause IL-10 upregulation (Figure 6A). Such a link is consistent with previous observations indicating that IL-10 upregulation in macrophages occurred in parallel with TGF-β increase and was blocked by a neutralising anti-TGF-β antibody. Also, concomitant to the astrocyte response, Gas6 could induce IL-10 upregulation in microglia, presumably via Axl, prior to TGF-β upregulation, and as a result, upregulated IL-10 could induce myelination, whilst TGF-β released from astrocytes would push OPCs towards a pathway that favours myelination (Figure 6B).

In conclusion, this study has shown the discrete expression of TAM receptors and their ligand Gas6 in various glial cell types and that Gas6 upregulates IL-10 and TGF-β expression in both optic nerve and mixed glial cell cultures. The activation of both Axl and Tyro3 in astrocytes appears to be necessary for the effect of Gas6, and the positive effect of Gas6 on myelination is mediated through IL-10 induction and release. Therefore, these findings support a prominent role for Gas6 as an anti-inflammatory molecule in conjunction with its pro-myelinating and pro-repair functions in the CNS.

## Supplementary Materials

The following are available online at https://www.mdpi.com/2073-4409/9/8/1779/s1, Figure S1: Basal endogenous IL-10 expression in TAM knockout and wildtype microglia. qRT-PCR analysis was performed on extracts from microglia primary cultures from wildtype (*n* = 11), Mer^−/−^ (*n* = 10), Axl^−/−^ (*n* = 6) and Tyro3^−/−^ (*n* = 1) mice, Figure S2: Effect of Gas6 on TGF-β expression in single-TAM knockout optic nerve cultures with or without Gas6 treatment. Values represent mean (±SEM, *n* = 3 experiments for Tyro3 knockout; *n* = 2 for Axl and Mer knockouts).
