# Supplementary material for: Gas6 Induces Myelination through Anti-Inflammatory IL-10 and TGF-β Upregulation in White Matter and Glia
Source: Cells. 2020 Jul 26;9(8):1779. doi: 10.3390/cells9081779 (PMC7465828; doi:10.3390/cells9081779)
Supplement: Supplementary file 1 [file cells-09-01779-s001.pdf]

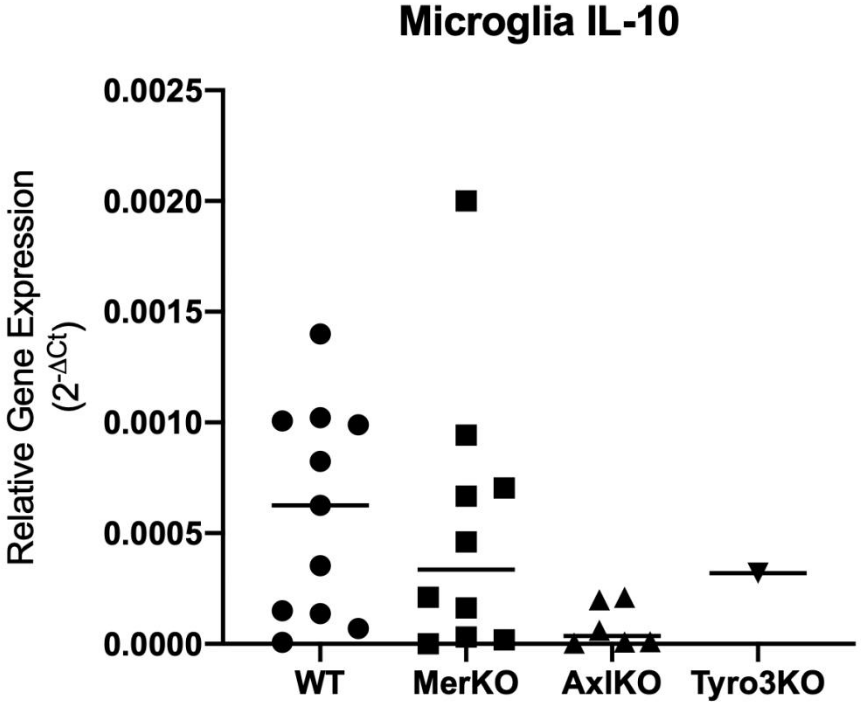

Figure S1. Basal endogenous IL-10 expression in TAM knockout and wildtype microglia. qRT-PCR analysis was performed on extracts from microglia primary cultures from wildtype (n=11), Mer-/- (n=10), Axl-/- (n=6) and Tyro3-/- (n=1) mice.

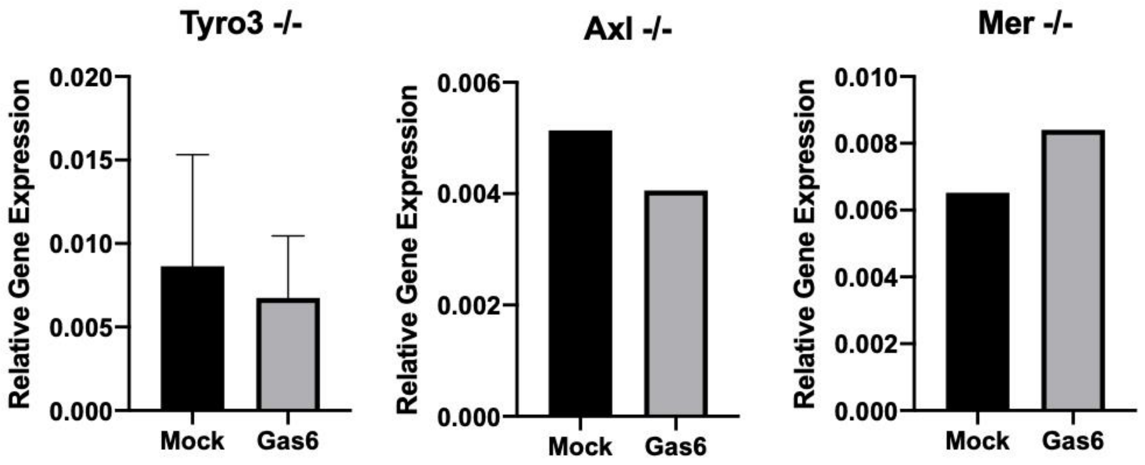

Figure S2. Effect of Gas6 on TGF-β expression in single TAM knockout optic nerve cultures treated with and without Gas6. Values represent mean (±SEM, n=3 experiments for Tyro3 knockout; n=2 for Axl and Mer knockouts).
